# Type 1 Diabetes in the Spanish Population: additional factors to Class II HLA-DR3 and -DR4

**DOI:** 10.1186/1471-2164-6-56

**Published:** 2005-04-20

**Authors:** Elena Urcelay, José L Santiago, Hermenegildo de la Calle, Alfonso Martínez, Julián Méndez, José M Ibarra, Carlos Maluenda, Miguel Fernández-Arquero, Emilio G de la Concha

**Affiliations:** 1 Immunology Department, Hospital Universitario San Carlos, Madrid, Spain; 2 Endocrinology Department, Hospital Ramón y Cajal, Madrid, Spain; 3 Diabetes Unit, Hospital Universitario San Carlos, Madrid, Spain; 4 Department of Paediatrics, Hospital Universitario San Carlos, Madrid, Spain

## Abstract

**Background:**

The Major Histocompatibility Complex is the main genetic contributor to susceptibility to type 1 diabetes (T1D); genome-wide scans have consistently mapped increased predisposition to this region. The highest disease risk has been associated with HLA-DR3 and HLA-DR4. In particular, the DR3-positive ancestral haplotype 18.2 was reported as highly diabetogenic. We aimed to corroborate whether this haplotype increases the susceptibility conferred by the DQ2-DR3 alleles in a Mediterranean population. We also searched for additional susceptibility factors to the classic DQ2-DR3 and DQ8-DR4.

**Results:**

Genetic MHC markers were analysed in a case-control study with 302 T1D patients and 529 ethnically matched controls. DR3-TNFa1b5 carrier rate was significantly higher in DR3-positive heterozygous T1D patients than in DR3-positive heterozygous controls (p = 0.0019; odds ratio OR [95% confidence interval CI] = 2.26 [1.3–3.93]). This data was confirmed analysing the allelic frequency, which includes the information corresponding to the DR3-homozygous individuals (p = 0.001; OR = 2.09) and by using the Arlequin software to check the DR3-positive haplotypes (p = 0.004;OR = 1.93). The present results provide strong evidence of a second susceptibility region in the ancestral haplotype 18.2 in the Spanish population.

Moreover, we searched for T1D susceptibility factors in addition to the MHC classical ones, within the DR2-DQ6/DR3-DQ2/DR4-DQ8 negative population. Several genetic markers in both MHC class II (DQA1*0101-DQB1*0501 [p = 0.007;OR = 2.81], DQA1*0201-DQB1*0202 [p = 0.03; OR = 2.35]) and III (TNFa2b1 [p = 0.01 OR = 2.74], BAT-2*2 [p = 0.004; OR = 3.19]) were found. These different alleles associated with T1D were not independent and we observed linkage disequilibrium among them leading us to describe two new risk haplotypes (DQA1*0101-DQB1*0501-TNFa2b1 and DQA1*0201-DQB1*0202- BAT-2*2). Finally, we studied a T1D susceptibility/protection marker located in extended class I, D6S2223; however, no association was observed in our population.

**Conclusion:**

Our results suggest that other associated MHC haplotypes might present susceptibility factors in loci different from HLA-class II and that the class II molecules are not necessarily the universal etiologic factor in every MHC haplotype.

## Background

Type 1 diabetes is a multifactorial autoimmune disease characterised by insulin deficiency, due to the T cell mediated destruction of pancreatic β-cells [1]. Among the genetic determinants of susceptibility, with more than 18 putative loci identified to date, a region in chromosome 6p21 (IDDM1) containing the Major Histocompatibility Complex (MHC) is the only one consistently associated with T1D in genome-wide screenings. The MHC spans approximately 4 Mb and consists of over 200 genes arranged in three subregions named class II, III and I. More than 90% of Caucasoid type 1 diabetic patients have at least one copy of the class II HLA-DR3 or DR4 allele, as compared to the 45% present in the general population and the genotype frequency of the DR3/DR4 heterozygote in T1D patients is 40% vs. 3% in controls [2]. In fact, the strongest susceptibility haplotypes described are DQB1*0201-DQA1*0501-DRB1*03 (DQ2-DR3) and DQB1*0302-DQA1*0301-DRB1*04 (DQ8-DR4), especially when both appear together in the genotype. However, not all HLA-DR3 or -DR4 positive haplotypes are equally predisposing [3,4]. This might suggest the role of other MHC loci responsible for modifying the susceptibility to diabetes conferred by class II genes, but their search has been difficult due to the strong linkage disequilibrium (LD) present in this chromosomal region. The term ancestral, extended haplotype was coined referring to continuous sequence derived with little, if any, change from an ancestor of all those now carrying all or part of the haplotype [5]. A published report stated that the DQ2-DR3-B18 AH 18.2 significantly increased risk compared to other haplotypes with the same class II alleles, but LD between markers hampered a more precise localisation of the presumed susceptibility gene [6]. Taking into account that this AH 18.2 is more frequent in Southern European populations, we decided to assess the possible role of other loci in the MHC region besides DQ-DR within this haplotype. We evaluated several genetic markers along the MHC in a case-control study with 302 Spanish T1D patients and 529 healthy controls. This study reproduced the especially strong association of the AH 18.2 with T1D in the Spanish population, supporting the existence of a second susceptibility locus within this haplotype. The present work extended the search to other T1D risk factors in the MHC besides DQ2-DR3/DQ8-DR4. Additional MHC haplotypes question the paradigm of class II genes as sole responsible for the association and open up the possibility that class III susceptibility factors would be also involved.

## Results

We first aimed to corroborate in the Spanish population the published increased risk to autoimmune diabetes of the AH 18.2, as compared to other DR3-positive haplotypes [6]. Microsatellites TNFa and TNFb, located in the untranslated region upstream of the LTA gene, exhibit extensive polymorphism and are good haplospecific markers. In order to identify the extended haplotype AH 18.2, we selected both microsatellites TNFab, which are conveniently close to HLA-B and easier to genotype than the latter. The microsatellites TNFa1b5 tag the AH18.2 defined by the characteristic alleles of five markers (D6S273*2, BAT2*2, TNFa1b5, MICA*4) in our families with IgA deficiency [7] and celiac disease [8]. These AH 18.2 markers were also confirmed by the computer program Arlequin, by linkage disequilibrium studies and by analysis of homozygous individuals. As we are dealing with patients and controls with no families at our disposal, the DR3-TNFa1b5 phase was uncertain. However, due to heavy linkage disequilibrium between these alleles, most of the times they will probably be in *cis*. This proportion can be estimated based upon the presence of TNFa1b5 in DR3-negative individuals: 8 carriers out of 109 DR3-negative T1D patients, and 13 carriers out of 376 DR3-negative healthy controls. These numbers yield allelic frequencies of TNFa1b5 on DR3-negative haplotypes of 7 and 3%, respectively. The assumption can therefore be made that, most of the time; a DR3 heterozygous sample carrying TNFa1b5 will in fact carry these alleles in the same haplotype.

Once the AH 18.2 was defined, we looked for the already described different distribution of this haplotype between T1D patient and control cohorts. Initially, we compared the carrier rate of TNFa1b5 among DR3-positive individuals. In T1D patients, the ratio of DR3 homozygous subjects out of the total DR3-positive individuals is much higher than the observed in controls (47 out of 193 vs. 8 out of 153 in controls; p = 1 × 10^-6^; OR = 5.83). This makes necessary to calculate the TNFa1b5 carrier rate exclusively in DR3 heterozygous individuals to avoid the cumulative effect of those individuals. When the TNFa1b5 carrier rate was compared, it was significantly higher in DR3-positive heterozygous T1D patients than in DR3-positive heterozygous controls (66 out of 143 vs. 33 out of 120; p = 0.0019; OR (95% CI) = 2.26 [1.3–3.93]). If we do not want to discard the information corresponding to the DR3-homozygous individuals, the comparison should be made counting allelic rather than phenotypic frequency and then normalising these counts by the total number of DR3-positive haplotypes present in the DR3-positive individuals (112/123 vs. 40/92, p = 0.001, OR = 2.09) (Table 1).

**Table 1:** Comparison of the TNFa1b5 allelic frequency in the DR3-positive T1D patient and control cohorts.

	*DR3-positive Individuals*	*Total haplotypes*	*DR3-positive haplotypes*	*TNFa1b5 alleles*	*TNFa2b3 alleles*
T1D	189	378	235	112	64
Controls	126	252	132	40	51

The results strongly suggest that the haplotype AH 18.2 is over represented among DR3 haplotypes. To formally elucidate whether this is the case, we observed that the allelic frequency of DR3-TNFa1b5 (AH 18.2) is higher than that of the DR3-positive abundant haplotype AH 8.1, which carries TNFa2b3, (112/40 vs. 64/51, p = 0.002, OR (95% CI) = 2.23 [1.29–3.86]). Moreover, when the allele frequencies of TNFa1b5 and TNFa2b3 were compared to the total of TNFab alleles excluding the two aforementioned, the AH18.2 showed significant difference (112/59 vs. 40/41, p = 0.01, OR = 1.95) whereas TNFa2b3, AH8.1, did not (64/59 vs. 51/41, p = 0.62, OR = 0.87). An alternative approach would consist of applying an expectation-maximisation algorithm, as the one implemented by the Arlequin program to the sample genotypes. Table 2 shows the DR3 three-loci haplotypes generated by this software sorted into TNFa1b5 positives, TNFa2b3 positives and other haplotypes. Again, TNFa1b5 is significantly different from other DR3 haplotypes, while TNFa2b3 behaves similarly to the rest.

**Table 2:** Frequency and number of DR3-positive haplotypes estimated by the Arlequin software.

	T1D (2n = 596)		Controls (2n = 1006)	
	Freq.	Haplotypes	Freq.	Haplotypes
DR3-Total	0.39432	235	0.13120	132
I. DR3-a1b5	0.17276	103	0.03802	38
II. DR3-a2b3	0.10244	61	0.04866	49
III. DR3-other	0.11912	71	0.04452	45

I vs. II+III. OR = 1.93 (1.19–3.13); p = 0.004.

I vs. II. OR = 2.18 (1.24–3.83); p = 0.004.

I vs. III. OR = 1.72 (0.98–3.01); p = 0.043.

II vs. III. OR = 0.79 (0.45–1.39); p = 0.38.

In our Spanish sample this DR3-TNFa1b5 positive haplotype was present in a 33% T1D patients (98/298) vs. 7% controls (37/504). The haplotypes ascertained by the Arlequin software yielded an AH 18.2 almost five times more frequent in the Spanish patient than control cohorts (Table 2). Moreover, almost one half (46%, 66/142) of the DR3 heterozygous diabetic patients vs. one forth (27%, 33/120) of the controls displayed a DR3-TNFa1b5 positive phenotype. On the contrary, in this subpopulation the distribution of the other main DR3-positive haplotype, AH 8.1, was similar between patients (43/142, 30%) and controls (47/120, 39%).

Therefore, these results confirmed the reported specific diabetogenic role of the AH 18.2 in a Mediterranean population and are compatible with published observations that established the presence of a second susceptibility gene in the AH 18.2 [6,9] possibly located in either class III or class I.

The second aim of this work was searching for additional T1D susceptibility factors to the classical DQ2-DR3 and DQ8-DR4 in the MHC region. Obviously, the subjects studied should belong to DR3- and DR4-negative populations to eliminate the underlying genetic risk attributable to these known risk factors and they were also stratified by DR2, to avoid the effect of this allele negatively associated with the disease. We tested the predisposition determined by alleles of the five genetic markers in HLA class III already studied (Table 3), and we also compared the distribution of the DQA1-DQB1 alleles (Table 4). Then, alleles TNFa10b4, DQA1*0501-DQB1*0301 and DQA1*0103-DQB1*0603 for protection, and TNFa2b1, BAT-2*2, DQA1*0101-DQB1*0501 and DQA1*0201-DQB1*0202 for susceptibility, displayed statistical significant difference when T1D patients and healthy controls were compared in the DR3, DR4 and DR2- negative population. These different alleles associated with T1D were not mutually independent and linkage disequilibrium was observed between TNFa2b1 and DQA1*0101-DQB1*0501 (p = 9 × 10^-5^; D' = 0.34), BAT-2*2 and DQA1*0201-DQB1*0202 (p = 4 × 10^-7^; D' = 0.36) and weak LD between TNFa10b4 and DQA1*0103-DQB1*0603 (p = 0.09; D' = 0.16). These results would suggest the presence of extended haplotypes increasing the risk to autoimmune diabetes in these conditions, although the exact location of the susceptibility gene remains undetermined. The allele 2 of microsatellite BAT-2 within the haplotype DQA1*0201-DQB1*0202 is the same present in the AH 18.2, and therefore a common susceptibility region could potentially be shared by both haplotypes. To test this possibility two adjacent markers to BAT-2 were analysed, one telomeric, MN6S1879 and other centromeric, N3-2-3. The MN6S1879*14 allele present in the AH 18.2 is the same found in LD with BAT-2*2 in the DR2-DQ6/DR3-DQ2/DR4-DQ8 negative patients (p = 7 × 10^-5^; D' = 0.33). Curiously, provided the extensive LD displayed by the AH 18.2, there are two different alleles (7 and 9) in the N3-2-3 marker corroborated in our deficit IgA and celiac disease families, in homozygous individuals and by Arlequin software. The allele N3-2-3*7 did not show LD with BAT-2*2 in the triple negative population (p = 0.87, D' = 0.014), but N3-2-3*9 (p = 0.052, D' = 0.26) showed a trend for association. Although this latter allele seemed to mark higher risk than N3-2-3*7, the difference did not reach statistical significance. There was indirect evidence supporting the role of the allele N3-2-3*9 in T1D susceptibility. In two DR3-TNFa1b5 homozygous control individuals there were three alleles N3-2-3*7 and one N3-2-3*9, while in nine T1D patients, there were nine alleles each. Moreover, in DR3-DQ2/DR4-DQ8 individuals carrying TNFa1b5, the allele present in N3-2-3 is 9 (41 out of 83 vs. 0 from 14, p = 0.0012) instead of 7 (25 out of 83 vs. 7 out of 12, p = 0.06, OR = 0.31). Of notice the different trend shown for the allele 9 conferring susceptibility and for the allele 7 conferring protection. However, one must be cautious in the interpretation of these data, as allele 9 is much more haplospecific for AH 18.2 than allele 7, which appear in several other haplotypes. Any ulterior analysis was prevented given the lack of DR3-DQ2/DR4-DQ8 control subjects displaying the AH 18.2.

**Table 3:** Comparison of the carrier rate of HLA class III genetic markers in the DR2-DQ6/DR3-DQ2/DR4-DQ8 negative diabetic and control cohorts.

Marker	Allele	T1D	Controls	OR	p
D6S273		n = 29	n = 251		
	1	6	30	1.92	0.15
	2	2	24	0.70	0.48
	3	18	121	1.76	0.16
	4	13	134	0.71	0.38
	5	10	114	0.63	0.26
					
BAT-2		n = 29	n = 263		
	2	20	108	3.19	0.004
	3	13	98	1.37	0.43
	7	10	127	0.56	0.16
	8	3	20	1.40	0.40
					
TNFab		n = 29	n = 234		
	1,5	1	10	0.80	0.65
	2,1	12	48	2.74	0.01
	2,3	1	6	1.36	0.56
	4,5	3	25	0.96	0.63
	5,5	4	21	1.62	0.29
	6,5	8	61	1.08	0.86
	7,4	9	51	1.61	0.26
	10,4	3	73	0.25	0.02
	11,4	2	21	0.75	0.52
					
MICA		n = 29	n = 233		
	4	8	45	1.59	0.30
	5	6	62	0.72	0.49
	5.1	5	74	0.45	0.11
	6	22	141	2.05	0.11
	9	12	78	1.40	0.40

**Table 4:** DQA1-DQB1 haplotypes in DR2-DQ6/DR3-DQ2/DR4-DQ8 negative T1D patients and controls.

Haplotype	T1D (n = 29)	Controls (n = 282)	OR	p
0101–0501	16	86	2.81	0.007
0102–0604	2	20	0.96	0.66
0103–0603	1	50	0.17	0.03
0201–0202	16	97	2.35	0.03
0201–0303	1	27	0.34	0.24
0401–0402	5	21	2.59	0.08
0501-0301	6	120	0.35	0.02

Finally, we tried to ascertain the role as T1D susceptibility/protection marker of the microsatellite located 4.9 Mb telomeric of DQ in extended class I, D6S2223. In Northern European countries allele D6S2223*3 was associated with a reduction in risk independent of the class II effect [9,10] and allele D6S2223*1 accounted for susceptibility to T1D [6]. These associations were not observed in Sardinia [11]. In our DR3, DR4 and DR2-negative study group we did not find any association (allele D6S2223*3 for protection: 28/29 in T1D patients vs. 223/234 in healthy controls, OR = 1.38, p = 0.61; allele D6S2223*1 for susceptibility: 0/29 vs. 2/234, p = 0.79). However, the limited number of patients after stratification decreases the statistical power of detection for a positive association in our population.

## Discussion

Our observations support the distinctive effect on type 1 diabetes susceptibility of the AH 18.2 among all the other DR3-positive haplotypes in the Spanish population. Both, allelic and phenotypic frequencies showed the increased risk conferred by the DR3-TNFa1b5 haplotype. Moreover, the different behaviour in terms of T1D predisposition of both DR3-positive main haplotypes, AH 8.1 and 18.2, argues in favour of a second susceptibility locus besides the standard class II molecules (DR and DQ) in the latter.

Previous studies reported the contribution to T1D susceptibility of a second region in the MHC besides DQ-DR genes. Among them, one concluded that this second region extended between HLA-B and BAT-3 in the highest risk DR3/DR4 genotype [12], while other mapped the critical region around the microsatellite D6S273 centromeric to TNF [13]. TNF-α has been identified as a diabetogenic effector molecule synergistic with IFN-γ in the induction of β-cell apoptosis [14-16] and therefore, it was of interest to consider the TNF region located in class III MHC as a candidate susceptibility locus. Transfected cells containing BAT1 promoter fragments from the 8.1 AH exhibited lower reporter activity compared to the 7.1 AH. Since the 7.1 and 8.1 AH are associated with resistance and susceptibility to T1D respectively, the observed effects are claimed to have significant implications for the pathogenesis of this disease [17]. We decided to study this region in our population. Our findings of BAT-2*2, TNFa2b1 and TNFa10b4 as genetic markers of the risk conferred by the DQ-DR locus agrees with those previous reports in terms of location. Most probably the etiological variant(s) will be in linkage disequilibrium with the BAT-2*2 allele. However, given the low recombination rate of the haplotype 18.2 (shown by the TNFa1b5 present in a DR3-negative context, see Table 3), and being BAT-2*2 a common allele, this microsatellite could mark a different susceptibility gene in the AH 18.2 and in other haplotypes. Clearly, the analysis of genetic markers in class II and class III provided an emerging pattern of susceptibility/protection haplotypes in addition to the classical DR3/DR4/DR2 determinants. In this sense, one could speculate that the traditional emphasis on class II alleles alone as responsible for increasing the T1D risk could be an oversimplification of a more complex reality where class III markers remained in the background.

Population studies provide invaluable information helping to map predisposition loci. A good example is the role of D6S2223 as a marker of autoimmune diabetes in several Nordic populations [9] which has been reproduced neither in Sardinia [11] nor in Spain (present data).

A large number of studies from different populations including the Spanish one [18] have indicated that common allelic variants at the class II HLA-DRB1, -DQA1 and -DQB1 loci are associated with T1D. These MHC class II molecules play an important role in the presentation of peptide antigens after intracellular processing to CD4 T- lymphocytes. The correlation between the relative T1D predisposition of class II alleles and the structure of their proteins has also been described [19]. However, the presence of HLA class II molecules alone does not by itself cause disease. The HLA transgenic mice develop diabetes when there is an islet "insult", even if the islet "insult" is, itself, not sufficient to precipitate disease [17].

## Conclusion

Our data prove that there are additional susceptibility/protection factors besides DR3/DR4/DR2 in HLA in the Spanish population and particular combinations of these minor risk factors could have important effects on susceptibility. The results of the present study provide evidence of the importance of the whole HLA in T1D risk gradient after stratification for the DR3, DR4 and DR2 determinants. The accent placed on class II in the canonical DQ2-DR3/DQ8-DR4 haplotypes is not necessarily extensive to other associated haplotypes where susceptibility might map to different MHC loci.

## Methods

302 T1D patients and 529 healthy unrelated controls, both groups composed of Caucasian individuals from the same Madrid area, were consecutively recruited after informed consent obtained from the indexed subjects or their parents when the patient was a child. The T1D patients were diagnosed at two hospitals in Madrid and the control subjects were collected among healthy blood donors. The age at onset for the T1D patients (150 men and 152 women) range 1–55 years old (median onset 15 years old). Diagnosis was based on patients' clinical features and laboratory data according to the criteria of the American Diabetes Association (ADA). All subjects were insulin-dependent at the time of the study. Ab-positivity was studied for GAD, IA-2, insulin and ICA. The protocol followed the principles expressed in the Declaration of Helsinki and was approved by the Hospital Ethics Committee.

Both patients and controls were genotyped for HLA-DQB1, DQA1 and DRB1 alleles [20] for 6 microsatellite markers (D6S273, BAT-2, TNFa, TNFb, MICA and D6S2223) as previously described [20-25]. Microsatellite alleles were ascertained using an ABI Prism™ 310 automatic sequencer (Applied Biosystems, Foster City, CA, USA). Each sample included an internal size standard (TAMRA 500, Applied Biosystems) to achieve a highly consistent measure.

Allelic distribution and frequencies of the haplotypes present in the control and diabetic cohorts were determined using a software for population genetics data analysis (Arlequin ver 2.000. Schneider S, Roessli D and Excoffier L. University of Geneva, Switzerland). Haplotypes were also corroborated by our previous findings in families affected by other immune related diseases, by LD studies in controls, and by the recent literature [6].

The frequencies of each marker allele in patients versus controls were compared by a standard chi-square test using a statistical software package (EPI-INFO v. 6.02; Center for Disease Control & Prevention CDC, U.S.A.) and a result was considered significant if p < 0.05. Statistics are performed upon the individuals with available data in the markers under comparison; therefore the final number could differ among different analyses. As our working hypothesis was to verify in the Spanish population the presumed special association with T1D of the characteristic alleles of the AH 18.2, there was no need for Bonferroni corrections in these initial comparisons.

## Abbreviations

T1D type 1 diabetes

MHC Major Histocompatibility Complex

HLA human leukocyte antigen

AH ancestral haplotype

LD linkage disequilibrium

TNF tumor necrosis factor

OR odds ratio

LTA lymphotoxin alpha.

Freq. frequency

## Authors' contributions

EU carried out the genotyping of some samples, participated in the statistical analysis and drafted the major part of the manuscript.

JLS carried out the genotyping of most of the patients and a great part of the controls, participated in the statistical analysis and drafted the manuscript. He participated in the design and coordination of the study.

HdlC made the diagnosis, and collaborated in the statistical analysis

AM carried out a great part of the genotyping of control samples and participated in the statistical analysis and drafted the manuscript

JM participated in the genotyping and recollection of samples, and in the statistical analysis.

JMI made the diagnosis, and collaborated in the statistical analysis

CM made the diagnosis, and collaborated in the statistical analysis

MFA participated in the design and coordination of the study and helped to collect the DNA samples.

EgdlC conceived of the study and helping its coordination, participated in the statistical analysis and drafted the manuscript

All authors have red and approved the final manuscript.
